# Hepatitis C in Haftanstalten: Behandlung und Barrieren auf dem Weg zu den Eliminierungszielen der Vereinten Nationen

**DOI:** 10.1007/s00103-023-03808-y

**Published:** 2023-12-28

**Authors:** Anna Dichtl, Daniela Jamin, Heino Stöver, Meryem Grabski, Bärbel Knorr

**Affiliations:** 1 Institut für Suchtforschung, Frankfurt University of Applied Sciences, Nibelungenplatz 1, 60318 Frankfurt am Main, Deutschland; 2 Deutsche Aidshilfe, Berlin, Deutschland

**Keywords:** Infektionskrankheit, Gefängnis, Behandlung, Barrieren, Drogenkonsum, Infectious disease, Prison, Treatment, Barriers, Drug use

## Abstract

**Hintergrund:**

Trotz genereller Fortschritte bei der Hepatitis-C-Behandlung in Deutschland ist unklar, inwieweit dies auch für bestimmte Schlüsselgruppen, wie etwa Inhaftierte, gilt.

**Methoden:**

In einer Kurzbefragung im Juni 2021 wurden die Justizministerien der Bundesländer über Datenerhebungs- und Diagnostikmethoden sowie die Prävalenz von Infektionen mit dem Hepatitis-C-Virus (HCV) und HCV-Behandlungen von Gefangenen in Deutschland befragt. Ergänzend dazu wurden Expert*inneninterviews zu Barrieren und bisher nicht genutzten Chancen der HCV-Behandlung in Haft geführt.

**Ergebnisse:**

Es zeigt sich, dass keine vollständige und flächendeckende Datenerhebung innerhalb der Justizministerien vorliegt. Präventionsmaßnahmen wie Opiatsubstitutionstherapie in Haft sind in allen teilnehmenden Bundesländern verfügbar. Spezifischere Angebote und Maßnahmen (z. B. Rasierer, Spritzentausch) finden sich nur vereinzelt und sind teilweise kostenpflichtig. Die Expert*innen zeigen auf, dass die Behandlung innerhalb der Justizvollzugsanstalten zwar grundsätzlich dem Äquivalenzprinzip nachkommt, aber die Zielgruppe schwerer zu erreichen ist.

**Fazit:**

Wichtig für eine erfolgreiche HCV-Eliminierung in Justizvollzugsanstalten sind eine flächendeckende Aufklärung und Beratung zur HCV-Behandlung, konsequente HCV-Testung und -Behandlung sowie Präventionsmaßnahmen zur Vermeidung von (Re)Infektionen.

## Abstract (en)

**Background:**

Despite general advances in the treatment of people with hepatitis C in Germany, it is unclear to what extent this also applies to specific key populations, such as prisoners.

**Methods:**

In a short survey conducted in June 2021, the ministries of justice in the federal states were asked about data collection and diagnostic methods as well as the prevalence of infections caused by the hepatitis C virus (HCV) and HCV treatment among prisoners in Germany. In addition, expert interviews were conducted regarding HCV treatment barriers and missed opportunities in prisons.

**Results:**

It is evident that there is no complete and comprehensive data collection within the ministries of justice. Prevention measures, such as opiate substitution therapy in prison, are available in all participating federal states. More specific offers and measures (e.g., razor and syringe exchange) are only found in a few cases and are sometimes subject to a charge. Experts point out that although treatment in prison generally complies with the equivalence principle: it is more difficult to reach the target group.

**Conclusions:**

Comprehensive education and counselling on HCV treatment, consistent HCV testing and treatment, and preventive measures to avoid (re-)infection are important for successful HCV elimination in prisons.

## Hintergrund

Seit Mitte der 1980er-Jahre ist der Justizvollzug zunehmend mit viralen und bakteriellen Infektionskrankheiten wie dem erworbenen Immunschwächesyndrom (Acquired Immune Deficiency Syndrome, AIDS), Hepatitiden (A, B und C), sexuell übertragbaren Krankheiten (Sexually Transmitted Diseases, STI) und Tuberkulose konfrontiert. Im Vergleich zur Allgemeinbevölkerung sind bestimmte Schlüsselgruppen, wie aktuell und ehemalig injizierende Drogenkonsument*innen (People Who Inject Drugs, PWID) sowie mit Hepatitis-C-Virus (HCV) und mit dem humanen Immundefizienz-Virus (HIV) Infizierte, in deutschen Haftanstalten deutlich überrepräsentiert [1]. Auch die Prävalenz von Hepatitis B unter PWID liegt bei 25 %, damit also 5‑mal höher als in der deutschen Allgemeinbevölkerung [2]. Die erste bundeseinheitliche Erhebung zur stoffgebundenen Suchtproblematik im Justizvollzug ergab, dass von 41.896 erfassten Gefangenen 44 % eine stoffgebundene Suchtproblematik zum Zeitpunkt des Haftantritts aufweisen [3]. Ein erheblicher Teil der Gefangenen setzt seinen Drogenkonsum auch in Haft fort [4]. Eine Studie zeigte, dass 30 % der Teilnehmenden mit Hafterfahrung auch innerhalb der Haft Drogen injizierten und dass 11 % der Teilnehmenden mit Drogenkonsum in Haft während der Inhaftierung mit dem injizierenden Konsum begannen [5]. Des Weiteren gehören Infektionserkrankungen zu den häufigsten Gesundheitsstörungen im Strafvollzug. Gefangene sind im Vergleich zur Allgemeinbevölkerung 48- bis 69-mal häufiger mit Hepatitis C und 7‑ bis 12-mal häufiger mit HIV infiziert [6]. Es wurde zudem eine Assoziation zwischen HCV-Infektion und Hafterfahrung für PWID festgestellt; mit steigender Haftdauer und Häufigkeit der Inhaftierungen stieg die HCV-Prävalenz [2]. Die Assoziation von HCV- bzw. HIV-Infektionen und Inhaftierung für PWID wurde in einer Studie in 17 europäischen Ländern bestätigt [7]. Menschen in Haft gehören somit zu den besonders vulnerablen Gruppen in Bezug auf HIV- und/oder HCV-Infektionen [8]. Die Anzahl der in Deutschland bisher im Justizvollzug antiviral behandelten Gefangenen ist verglichen mit der anzunehmenden Prävalenz niedrig [9].

Die Vereinten Nationen (UN) haben nun unter Ziel 3.3 erstmalig auch die Beendigung von HIV und HCV als Bedrohung der öffentlichen Gesundheit in die nachhaltigen Entwicklungsziele (Sustainable Development Goals, SDGs) der Agenda 2030 aufgenommen [10].

Festgeschrieben wurde, dass bis zum Jahr 2030 90 % aller HCV-Infizierten diagnostiziert und 80 % der Behandlungsbedürftigen behandelt sind. Des Weiteren wird eine Reduktion der HBV- und HCV-Inzidenzen um 90 % sowie der HBV- und HCV-assoziierten Todesfälle um 65 % im Vergleich zu 2015 angestrebt [10]. In Deutschland wurde 2016 als Rahmen für die Umsetzung der Ziele der Agenda 2030 die deutsche Nachhaltigkeitsstrategie verabschiedet und im Anschluss daran für eine nachhaltige Eindämmung von HIV, Hepatitis B und Hepatitis C vom Bundesministerium für Gesundheit (BMG) die „Strategie zur Eindämmung von HIV, Hepatitis B und C und anderen sexuell übertragbaren Infektionen bis 2030“ (BIS2030) vorgelegt [11].

Seit der Verabschiedung der Agenda 2030 sind 6 Jahre, seit der Zulassung der neuesten Generation direkt wirkender antiviraler Medikamente (Direct Acting Antivirals, DAAs) bereits 8 Jahre vergangen. Generell wird bei einem Verdacht auf HCV das Blut des/der Patient*in zunächst auf HCV-Antikörper getestet. Können diese nachgewiesen werden, wird mittels Virus-RNA-Test geprüft, ob es sich um eine ausgeheilte oder akute Infektion handelt [12]. Die Therapie von HCV dauert heute – weitestgehend nebenwirkungsfrei und bei > 95 % Heilungschancen – bei Patient*innen im nicht-zirrhotischem Stadium durchschnittlich 12 Wochen [13, 14] und bei Patient*innen im zirrhotischem Stadium 14 Wochen [15]. Zwölf Wochen nach Ende der Therapie wird das Blut noch einmal auf HCV-RNA getestet, um einen dauerhaften Therapieerfolg festzustellen [12].

Neueste Daten belegen eine günstige Preisentwicklung bei DAAs, welche bei ihrer Zulassung noch sehr kostenintensiv waren. Inzwischen sind die Kosten einer HCV-Therapie durch günstigere Medikamente und eine kürzere Behandlungsdauer deutlich gesunken [15].

Die Voraussetzungen für eine tatsächliche Eliminierung von HCV sind daher grundsätzlich sehr gut, dennoch scheint sie in Deutschland vor allem mit Blick auf bestimmte Schlüsselgruppen unrealistisch [16, 17]. Vor diesem Hintergrund ergibt sich auch die Frage nach der aktuellen Situation in Justizvollzugsanstalten (JVAen) in Deutschland.

Der vorliegende Artikel gibt einen Überblick über die aktuellen Datenerhebungs- und Diagnostikmethoden der Bundesländer sowie, soweit vorhanden, die aktuellen HCV-Behandlungs- und Behandlungsabschlussprävalenzen von Gefangenen in Deutschland, die mithilfe von Kurzbefragungen der Justizministerien erhoben wurden. Im Anschluss daran werden die durch Expert*inneninterviews erhobenen Barrieren und Chancen für die HCV-Behandlung in Haft dargestellt, vor dem Hintergrund der Eliminierungsziele diskutiert und abschließend Handlungsempfehlungen für den Zeitraum bis 2030 formuliert.

## Methoden

### Kurzfragebogen zur Erhebung der HCV- und Behandlungsprävalenzen bei Inhaftierten

Die Befragung hatte das Ziel, eine aktuelle Übersicht über die HCV-, Behandlungs- und Behandlungsabschlussprävalenzen bei Inhaftierten innerhalb des Justizvollzugs zu erhalten. Sie wurde im Juni 2021 verschickt. Der Kurzfragebogen enthielt hauptsächlich geschlossene, quantitative und einige offene Fragen zu folgenden Schwerpunkten:

1. Frageblock „HCV-Testung und Prävalenzen“: Anzahl der Testungen und deren Ergebnisse, Art der Testung (Polymerase-Ketten-Reaktion [PCR] oder Antikörpertest), Anzahl von Behandlungsbeginnen und -abschlüssen sowie Rahmenbedingungen und systematische medizinische Test- und Behandlungsangebote. Ziel war es, einen Überblick über Erhebungs- und Diagnostikmethoden sowie aktuelle Prävalenzen in den JVAen der verschiedenen Bundesländer zu erhalten.
2. Frageblock „HCV-Prävention“: gezielte Fragen zu den zur Verfügung stehenden Präventionsmaßnahmen (drogenbezogene Präventionsmaßnahmen, z. B. Spritzentausch, Desinfektionsmöglichkeiten, sowie allgemeine Präventionsmaßnahmen, z. B. Kondome, Rasierer, Nagelscheren). Abgefragt wurden zum einen die Verfügbarkeit innerhalb der JVAen in den Bundesländern allgemein und zum anderen, inwieweit die Maßnahmen für die Inhaftierten kostenfrei sind.

Der Fragebogen wurde mit einem dazugehörigen Anschreiben im Juni 2021 per Post an alle 16 Justizministerien in Deutschland versendet. Zudem erhielten alle einen Link zur Onlinebefragung, sodass es im Ermessen der Bundesländer lag, ob sie die Fragen in Papierform oder online beantworten. Das Anschreiben enthielt die Bitte, bei fehlenden Daten, die ggf. nicht für das gesamte Bundesland vorliegen, den Fragebogen und den Link an die einzelnen JVAen weiterzuleiten. Der initiale Beantwortungszeitraum von 6 Wochen wurde 3‑mal auf insgesamt 24 Wochen verlängert. Die Kommunikation erfolgte ausschließlich durch die Justizministerien der Bundesländer (nicht durch einzelne JVAen). Die Fragebögen wurden mit der Software Evasys ausgewertet.

### Expert*inneninterviews zu HCV in JVAen

Um einen Überblick über die Chancen und Barrieren einer HCV-Behandlung im Justizvollzug zu erhalten, wurden 5 leitfadengestützte Expert*inneninterviews geführt. Als Expert*innen kamen Personen infrage, die durch ihre Arbeit mit der Behandlung von HCV in Haft vertraut sind (z. B. Ärzt*innen und Wissenschaftler*innen). Zur Strukturierung der Interviews wurde ein Leitfaden entwickelt. Dieser wurde offen gestaltet und beinhaltete folgende Fragenblöcke:

1. HCV-Eliminierungsziele,
2. HCV-Situation in Haft – allgemein,
3. HCV-Situation in Haft – Diagnostik und Testung,
4. HCV-Situation in Haft – Behandlung,
5. HCV-Situation in Haft – Prävention,
6. Rolle der JVAen für die Eliminierungsziele.

Für die Durchführung der Expert*inneninterviews wurden zunächst 10 ausgewählte Expert*innen telefonisch und per E‑Mail kontaktiert. Hiervon erklärten sich schlussendlich 5 zu den Interviews bereit. Die Interviews wurden bedingt durch die Einschränkungen im Rahnen der COVID-19-Pandemie telefonisch im Zeitraum von Juli 2021 bis Dezember 2021 geführt und zur späteren Transkription aufgezeichnet. Die anonymisierte Auswertung erfolgte angelehnt an die qualitative Inhaltsanalyse nach Mayring mit der Bearbeitungssoftware MAXQDA [18]. Im Zentrum der Auswertung standen zuvor gebildete Kategorien, die sich an den Leitfragen des Interviews und den Fragestellungen der Untersuchung orientierten.

## Ergebnisse

### HCV-Diagnostik, -Prävalenzen und -Behandlung in Haft – Ergebnisse Befragung Justizministerien

**Figure 1:**
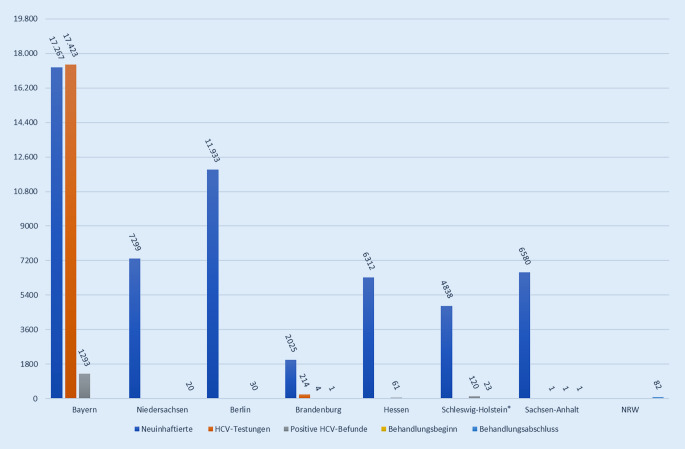

Die Datenlage zu HCV innerhalb der JVAen ist lückenhaft. Von den 16 kontaktierten Justizministerien sendeten 8 den Fragebogen zurück: Bayern, Berlin, Brandenburg, Hessen, Niedersachsen, Nordrhein-Westfalen (NRW), Sachsen-Anhalt und Schleswig-Holstein. In keinem dieser Bundesländer lagen vollständige Daten zu HCV-Testung, Testergebnissen, Behandlungsbeginnen sowie Behandlungsabschlüssen vor (Abb. 1). Keine der angefragten Daten sind bundeseinheitlich erfasst. Laut Aussage der Bundesländer werden Daten, für die keine Angabe erfolgte, nicht erfasst oder müssten aufwendig über die medizinischen Akten ausgewertet werden. Aufgrund der lückenhaften Datenlage ist es daher nicht möglich, HCV-Prävalenz, -Testung und -Behandlung umfassend darzustellen.

#### HCV-Diagnostik (Screening und Testung)

In 6 der 8 Bundesländern erhalten Inhaftierte im Rahmen der medizinischen Aufnahmeuntersuchung oder in den ersten 4 Wochen regelhaft ein HCV-Testangebot. Während der Inhaftierungszeit werden in Bayern, Berlin und NRW regelhaft weitere HCV-Testangebote gemacht, lediglich in Bayern bekommen Inhaftierte regelhaft ein HCV-Testangebot im Rahmen der Entlassung/Abschlussuntersuchung. Nur in Bayern und Brandenburg kann nachverfolgt werden, bei wie vielen Personen tatsächlich ein HCV-Test erfolgt ist.

#### Prävalenzen von HCV, HCV-Behandlungen und -Behandlungsabschlüssen in Haft

Auf Grundlage der erhobenen Daten sind keine Aussagen zu Behandlungen und Behandlungsabschlüssen möglich. Die meisten Bundesländer haben die Zahl der Neuinhaftierten dokumentiert, nicht jedoch die Anzahl der Testungen, Behandlungen und Behandlungsabschlüsse. Nur in Bayern und Brandenburg wurde die Anzahl der Testungen und positiven Ergebnisse dokumentiert. Hieraus ergibt sich, dass in Bayern 1293 von 17.423 durchgeführten Testungen (7,5 %) positiv waren. In Brandenburg waren 4 von 214 Testungen positiv (1,8 %). Allerdings ist diese Positivenquote aufgrund der relativ kleinen Testanzahl nur bedingt aussagekräftig. Auch aus Bayern, wo detailliert die Anzahl der Neuinhaftierungen, Testungen und positiven Ergebnisse dokumentiert wird, liegen keine Daten zu Behandlungen und Behandlungsabschlüssen vor.

#### HCV-Präventionsmaßnahmen und -angebote

**Figure 2:**
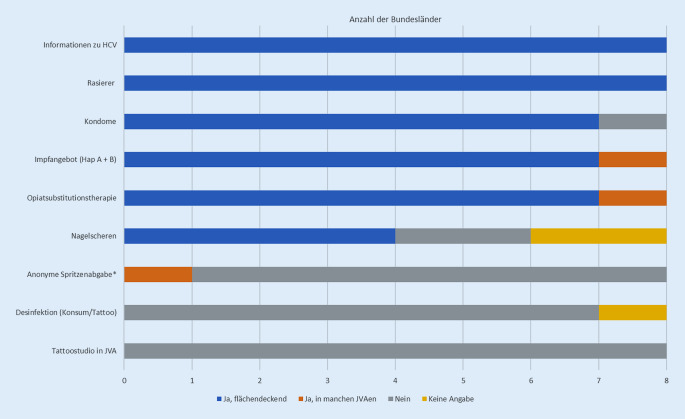

In allen teilnehmenden Bundesländern waren Informationen zu HCV, Rasierer als allgemeine Präventionsmaßnahme sowie HAV- und HBV-Impfangebote verfügbar. Letzteres liegt in Hessen im Ermessen der Anstaltsärzt*innen. Eine Opiatsubstitutionstherapie ist in allen teilnehmenden Bundesländern verfügbar, in 7 davon flächendeckend (in Hessen nicht flächendeckend). Kondome sind in 7 Bundesländern flächendeckend verfügbar, in Brandenburg nicht verfügbar. Nagelscheren bzw. -knipser sind in NRW, Schleswig-Holstein, Hessen und Brandenburg verfügbar. Eine anonyme Spritzenabgabe erfolgt in Deutschland nur in einer JVA in Berlin. Desinfektionsmöglichkeiten für beispielsweise Konsumutensilien oder Tätowiermaschinen sowie ein Tattoostudio innerhalb der JVA stehen in keinem der teilnehmenden Bundesländer zur Verfügung (Abb. 2).

**Figure 3:**
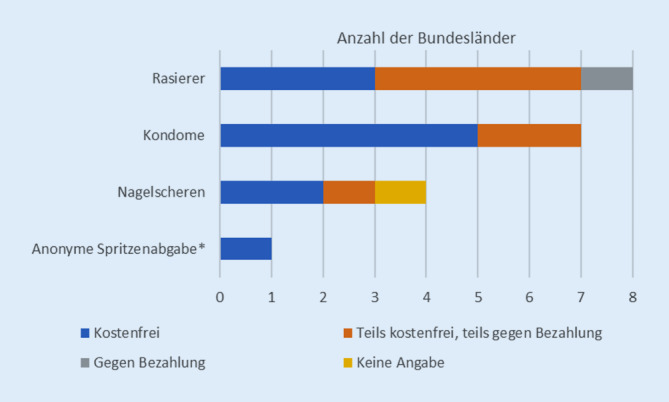

Die in den teilnehmenden Bundesländern flächendeckend zur Verfügung stehenden Rasierer sind in 3 Bundesländern für Inhaftierte kostenfrei, in einem Bundesland gegen Bezahlung und in 4 Bundesländern nur teilweise kostenfrei zugänglich. Kondome sind in 5 der 7 Bundesländern kostenfrei und in den anderen 2 nur teilweise kostenfrei erhältlich. Nagelscheren stehen in 2 Bundesländern kostenfrei und in einem Bundesland nur teilweise kostenfrei zur Verfügung. Die anonyme Spritzenabgabe in einer Berliner JVA ist über einen Automaten für Inhaftierte kostenfrei (Abb. 3).

Die Angaben der Bundesländer beziehen sich auf die theoretische Verfügbarkeit der genannten Angebote/Maßnahmen. Aus den Daten geht nicht hervor, inwieweit die Inhaftierten auch tatsächlich Zugang zu den Angeboten haben. Die Opiatsubstitutionstherapie ist z. B. grundsätzlich im Großteil der JVAen in Deutschland verfügbar, die Substitutionsquote unter den Personen, die die Kriterien zu einem Behandlungsbeginn erfüllen, liegt jedoch lediglich bei 23,9 % [3].

### HCV-Testung, -Behandlung und -Prävention in Haft – Ergebnisse der Expert*inneninterviews

#### Exkurs in die Behandlungspraxis in Haft

Fünf (ausschließlich Ärzt*innen) erklärten sich zu einem Interview bereit. Die Behandlungspraxis in Haft stellt sich in der Vorgehensweise bei allen befragten Expert*innen ähnlich dar. Zu Beginn erfolgt eine HCV-Testung bzw. Diagnose durch die medizinische Abteilung der JVA. Auch wenn die Aufnahmeuntersuchung in JVAen obligatorisch ist, findet die Testung nach Erfahrung der Expert*innen immer auf freiwilliger Basis statt. Nach einer positiven Diagnose erfolgt ein Beratungsgespräch mit den Patient*innen. Im Anschluss erfolgt zum einen ein Eintrag im sogenannten C‑Bogen in der Gefangenenpersonalakte, dass Infektionsgefahr bei Blutkontakt besteht. Dieser Eintrag ist für alle Bediensteten der JVA einsehbar. Zum anderen erfolgt eine Überweisung an eine infektiologische Ambulanz. Auf Basis der für die Therapie notwendigen weitergeleiteten Informationen (Genotyp, Viruslast etc.) sowie einer weiteren Untersuchung in der infektiologischen Ambulanz erfolgt die Entscheidung für oder gegen eine Behandlung. Die Betroffenen können die Therapie zu diesem Zeitpunkt immer noch ablehnen. Die Therapieempfehlung wird im Anschluss der JVA übermittelt, welche dann ggf. über den medizinischen Dienst die Medikamente bestellt. Nach dem anberaumten Behandlungszeitraum erfolgt eine Nachkontrolle in der infektiologischen Ambulanz.

Laut den Expert*innen spielen auch weitere Kriterien für einen Behandlungsbeginn in Haft eine Rolle. So wird die Therapieentscheidung auch unter Berücksichtigung von Lebensstil, Resthaftdauer, aktueller Situation usw. getroffen. Daraus folgt, dass insbesondere bei kurzen Haftstrafen oder besonderen Haftformen (Untersuchungshaft) sowie bei geringeren Erfolgschancen trotz Indikation keine Behandlung empfohlen wird. Ein*e Expert*in nannte zudem das Budget eines Bundeslandes als möglichen Einflussfaktor für eine Behandlungsentscheidung und die Vorgehensweise der medizinischen Abteilung der JVA.

### Barrieren und Chancen der Testung und Behandlung im Justizvollzug

#### Barriere: Finanzierung

„Wir wissen ja, das wird zwar oft bestritten, aber … Budgets gibt es dafür und die haben gesagt, wir können so viele behandeln so viel Geld wie wir haben …“ (Interview HCV_3).

Die Expert*innen sind sich einig, dass die Finanzierung eine der Hauptbarrieren für flächendeckende HCV-Testung und -Behandlung in Haftanstalten darstellt. Sie schätzen, dass die Anzahl der Tests und vor allem die Anzahl der Behandlungen steigen würden, wenn die Kosten von den gesetzlichen Krankenkassen (GKV) und nicht den finanziellen Mitteln der Justiz getragen werden würden. Aus ärztlicher Perspektive wird die Situation äußerst kritisch gesehen, da dadurch nicht alle indizierten Behandlungen durchgeführt würden.

„Es gibt natürlich … immer diese Ideale, die man anstrebt, wie das WHO-Ziel. Die andere Frage ist natürlich … immer: Wer zahlt? Das darf man … auch nicht außer Acht lassen. Das kann nicht das Argument sein, jemandem, der eine Behandlung dringend bedarf, die zu verweigern. … Auch da habe ich schon von einer Justizvollzugsanstalt gehört, wo die Anstaltsleitung dem Anstaltsarzt verboten hat, weitere Hepatitis-C-Medikamente zu bestellen, weil sie ja so teuer wären. Dann wäre das Budget aufgebraucht …, was ich tatsächlich … äußerst fragwürdig finde“ (Interview HCV_4).

„… dass eben bewusst deshalb weniger getestet wird, um die Kosten nicht so in die Höhe zu treiben. … Ich denke, dass eine ernsthafte Behandlung tatsächlich nur bei dem stattfindet, … [bei dem] … regelmäßig, eben bei Substitution, das Blut auch untersucht wird“ (Interview HCV_5).

Zu geringe medizinische Budgets im Justizvollzug führten daher möglicherweise dazu, dass JVA-Ärzt*innen nicht immer proaktiv handeln und von sich aus HCV-Testungen und -Behandlungen anbieten – auch wenn diese indiziert seien.

„… das ist auch das Problem, warum in Haft nicht proaktiv gehandelt wird von den Medizinern [sic!], also dass man tatsächlich sehr, sehr, sehr zurückhaltend ist von ärztlicher Seite aus, um auf Leute zuzugehen. Und deshalb glaube ich, wird so wenig behandelt und man könnte, wenn die Ärzte proaktiver wären, deutlich mehr Menschen behandeln, als … jetzt behandelt werden“ (Interview HCV_3).

#### Barriere: Stigmatisierung

„… was man ja auch weiß, dass natürlich auch die Bediensteten über positive Testergebnisse … informiert werden … [und] es die Vermerke auf der Akte gibt mit rotem Punkt … Und … dass es mal auch Stigma geben kann, wenn sich Leute testen lassen …, ist sicherlich auch nochmal ein Problem. … Ich glaube, dass man das Thema Stigmatisierung von Menschen mit Infektionserkrankung in Haft angehen muss. … Wir wissen ja, dass immer noch bekannt wird – und zwar nicht nur im medizinischen Dienst, sondern darüber hinaus –, dass wenn Leute eine übertragbare Infektion haben, HIV oder auch Hepatitis, … es dann besondere Hinweise im Umgang für das Personal usw. … gibt. Und das wissen die Leute natürlich auch und das ist sicherlich auch ein Problem, dass sie sich nicht testen lassen“ (Interview HCV_4).

Als weitere Barriere wird von den Expert*innen die fortwährende Stigmatisierung von Menschen mit HCV betrachtet. Diese hätten einerseits Angst vor der Verurteilung und Diskriminierung durch Mitgefangene, andererseits vor Einschränkungen im Haftalltag. So berichteten Inhaftierte immer wieder davon, nach einem positiven Testergebnis Einschränkungen, z. B. bei der Arbeitsplatzwahl in Haft, zu unterliegen. Dies führe dazu, dass sie sich bei einer nächsten Inhaftierung nicht mehr testen ließen und habe auch abschreckende Wirkungen auf Mitgefangene, die sich aus Angst vor Stigmatisierung gar nicht erst testen ließen.

„Da kann man auch in den Brief reinschreiben: Aus medizinischer Sicht spricht überhaupt nichts dagegen mit einer Hepatitis C hier mal die Kartoffeln zu schälen … Ja, aber das wird in den Anstalten oft noch so gehandhabt, dass gesagt wird, ‚Hepatitis C und HIV geht nicht in die Küche.‘ Das muss keinen Sinn machen, aber das ist dann nun mal so“ (Interview HCV_3).

#### Barriere: Unwissenheit

Sowohl die Unwissenheit über die Übertragungswege von HCV als auch über neuere Therapiemethoden werden als weitere Barrieren für erfolgreiche Behandlungen ohne Reinfektionen genannt.

„… Also dass viele gesagt haben, ‚… [eine Interferon-Behandlung] hatte ich schon mal vor zehn Jahren, … das mache ich nicht nochmal.‘ Also auch da wenig Aufklärung, wenig Wissen, … dass es eine neue Form, eine andere Form der Behandlung gibt. … Ich glaube auch nicht, dass es eine [große] Ablehnung geben würde, wenn überall bekannt wäre, dass es … eben nicht mehr die Interferon-Behandlung [gibt]“ (Interview HCV_5).

#### Barriere: nicht zielgruppengerechte Testangebote

„… Was in meinem Dafürhalten eine absolut ungeeignete Situation … ist, weil für den Menschen ändert sich … ja gerade alles. Der muss sich in die Anstalt eingewöhnen, der möchte eigentlich erstmal wissen ‚wo krieg ich meine Zigaretten her?‘, oder ähnliche Grundbedürfnisse“ (Interview HCV_2).

Ein Testangebot bei Haftantritt halten die Expert*innen für nicht zielführend, da zu diesem Zeitpunkt andere Probleme eine große Rolle für die Gefangenen spielen würden, die diese zunächst verarbeiten müssten.

„… wir haben … sehr, sehr viele hochindividuelle Einzelfälle mit multiplen Problemlagen. Den interessiert, wenn er hier bei uns ankommt, ‚was wird aus meinem Hund?‘, aber nicht ‚was wird aus meiner Leber?‘. Die zweite Frage ist, ‚wie komm ich an Stoff?‘ Aber immer noch nicht ‚was wird aus meiner Leber?‘ Die dritte Frage ist ‚wie komm ich hier schneller raus?‘ und das dauert eine ganze Weile, bis dann jemand sagt ‚nee, jetzt will ich aber wirklich mal‘. [Es] … sind wenige, die dann sagen ‚jetzt will ich aber wirklich mal einen Schlussstrich ziehen und das alles gut machen‘, sondern da sind andere Kernfragen am Anfang“ (Interview HCV_1).

Es müsse ein auf die Gefangenen abgestimmtes Angebot geben, dass in der Haftzeit mehrmals angeboten wird. Eine Grundvoraussetzung sei dabei auch, dass die individuellen Problemlagen der Gefangenen parallel adäquat bearbeitet würden, um diesen eine Chance einzuräumen, sich auf eine Behandlung einzulassen.

#### Chancen: externe Beratung und Testung

„Ich bin immer wieder ein Verfechter [dafür,] noch mehr externe Partner … reinzuholen und aufzuklären. Wir würden uns wünschen, dass wir im Zweifelsfall auch über HCV-Schnelltestkonzepte weiterkommen, dass … auch die HCV-Testung als Laientestung durchgeführt werden kann …, wie wir das bei der AIDS-Testung … schon haben. Das wäre ein wichtiger Schritt, um dann … auch über externe Berater diese Thematik aufzumachen und [ein] ähnlich gutes Setting zu bekommen, wie wir es im HIV-Bereich … bereits … haben“ (Interview HCV_1).

„… Auch wenn sich die Justizen oder Ambulanzen überfordert fühlen, finde ich auf jeden Fall unabhängige Gesundheitsberatung in Haftanstalten [notwendig]. Also im Zweifel externe Leute in die Anstalten holen“ (Interview HCV_5).

Die momentane Handhabung von HIV und Aids könnte ein Vorbild für den Umgang mit HCV sein: Um Stigmatisierungen zu vermeiden und aktuelle Lücken in der HCV-Prävention, -Beratung und -Testung zu füllen, sollten vor allem häufiger und mehr Informationsveranstaltungen, Beratungen und Testungen von externen Partner*innen angeboten werden – zum Beispiel auch Schnelltests, die von Laien durchgeführt werden können.

#### Chancen: Abgabe steriler Konsumutensilien

„[Ein Spritzentausch] wäre ganz sinnvoll, weil es gibt … Leute, die sagen ‚… ab und zu spritz ich in Haft und dann benutzen wir natürlich Spritzen, die andere auch schon mal in den Fingern gehabt haben, weil man da schlecht drankommt‘. Das ist natürlich fatal! …“ (Interview HCV_3).

Auch die Abgabe steriler Konsumutensilien an Inhaftierte wird von den Expert*innen als sinnvoll erachtet, um Infektionen zu vermeiden.

#### Exkurs Äquivalenzprinzip

Nach dem Äquivalenzprinzip soll die medizinische Versorgung der Gefangenen den außerhalb des Vollzugs erprobten und bewährten Standards entsprechen [18], was insbesondere auch in Bezug auf eine HCV-Behandlung in Fachkreisen diskutiert wird. Basierend auf gesetzlichen Grundlagen entsteht der Anspruch einer gleichwertigen Behandlung innerhalb des Vollzugs, die denen der Leistungen der GKV entspricht. Auf das Äquivalenzprinzip wurde im Rahmen der Expert*inneninterviews mehrfach Bezug genommen. Die Expert*innen stimmen überein, dass die Versorgung im Justizvollzug bereits nahezu äquivalent zu den Versorgungsangeboten außerhalb der Haft zu betrachten sei. Allerdings sei die Patient*innengruppe innerhalb der Haft nicht mit Patient*innen außerhalb der Haft zu vergleichen und bringe daher auch in der Behandlung Spezifika mit sich. Grundsätzlich biete die Haft gute Rahmenbedingungen für eine erfolgreiche Behandlung, da die Patient*innen vor Ort seien und aktiv auf sie zugegangen werden könne. Die Gruppe sei jedoch insbesondere bei Haftantritt mit individuellen Problemlagen konfrontiert und daher oftmals nicht bereit, sofort über eine HCV-Behandlung zu entscheiden. Diese Problemlagen müssten zunächst priorisiert und differenziert betrachtet werden. Ein weiterer Aspekt seien die vollzuglichen Rahmenbedingungen, sodass eine HCV-Behandlung aus medizinischer Sicht durchaus notwendig sein kann, aber die Behandlung auch zweckmäßig, also mit einem „vertretbaren Risiko zum Erfolg“ führen müsse. Sowohl Resistenzbildung als auch die wirtschaftlichen Nachteile bei einem Abbruch der Behandlung müssen beachtet werden. „Zweckmäßigkeit“ könne daher bedeuten, dass Personen in Haft aufgrund der Länge der Reststrafe oder anderen individuellen Faktoren (drohende Abschiebung, Untersuchungshaft etc.) kein Behandlungsangebot erhalten.

„Draußen kriegen alle Therapie, also kriegen drinnen alle Therapie. Draußen kriegen nicht alle Therapie, aber drinnen kriegen leider noch weniger Therapie. Weil es eine andere Gruppe ist, weil andere Rahmenbedingungen gelten, aber sie müssen alle das Äquivalent angeboten bekommen können. … Was [wir] hier wirklich mal hervorheben müssen ist …, dass wenn wir die gleichen Angebote und [den gleichen Zugang zur Therapie] in Haft schaffen, wir einfach aufgrund der Menschen andere Ergebnisse erzielen. Das ist das Entscheidende, … was wir rausarbeiten müssen … Wie können wir vielleicht zum Erreichen dieser Ziele, Spezialprogramme aufsetzen, die dann auch noch politisch und haushalterisch getragen werden?“ (Interview HCV_1).

Die Äquivalenz in Bezug auf eine HCV-Behandlung an sich ist laut den befragten Expert*innen zwar gegeben. Allerdings multiplizieren sich die Probleme, die auch außerhalb der Haft bestehen, bestimmte Schlüsselgruppen in Haft zu erreichen. Außerhalb der Haft würden viele Patient*innen nicht selbstständig, sondern nur über Angebote der Drogenhilfe medizinische Unterstützung aufsuchen. Diese Hilfe sei in Haft noch geringer ausgeprägt. Somit sei die Äquivalenz in Bezug auf HCV-Behandlung prinzipiell gegeben, aber nicht unbedingt in Bezug auf niedrigschwellige Angebote.

„[Es] ist ja ganz selten der Fall, dass jemand von der Straße weg zu irgendeinem Arzt geht und sagt, ‚ich will hier behandelt werden‘. Sondern es geschieht immer mit Hilfe und die ist natürlich in Haft deutlich geringer ausgeprägt als … draußen. … Wenn wir nicht so genau auf die Leute immer wieder … zugehen würden, hätten wir nur einen Bruchteil der Behandelten“ (Interview HCV_4).

## Fazit und Handlungsansätze

Der Justizvollzug bietet durch die obligatorische Aufnahmeuntersuchung durch Vollzugsärzt*innen sowie die Möglichkeit der regelmäßigen Beratung grundlegend optimale Rahmenbedingungen für eine HCV-Testung und -Behandlung. Insbesondere die Zielgruppe der Drogengebrauchenden, der oft eine geringe Compliance zugeschrieben wird, könnte in einer geschlossenen Institution gut erreicht werden [19]. Insgesamt ist jedoch zunächst die Datenlage zu HCV-Testungen, -Testergebnissen, Behandlungsbeginnen sowie Behandlungsabschlüssen in deutschen JVAen zu verbessern. In keinem der hier teilnehmenden 8 Bundesländer lagen vollständige Daten hierzu vor.

Die Ergebnisse zeigen weiterhin, dass im Umgang mit HCV (Prävention, Testung, Behandlung) die möglichen Potenziale des Justizvollzugs bisher nicht vollständig genutzt werden. Aus den Expert*inneninterviews können Ansätze für Handlungsempfehlungen aufgezeigt werden, die zu einer besseren Versorgung führen können. Im Rahmen der Prävention sollte die Vermeidung einer HCV-Infektion adressiert werden, z. B. auch durch die anonyme Vergabe von Konsummaterialen. Weiterhin sollten Aufklärungskampagnen bezüglich der Ansteckungswege und Behandlungsoptionen für Mitarbeiter*innen und Inhaftierte durchgeführt werden. Im Kontext der Testung sollte allen Inhaftierten zu verschiedenen Zeitpunkten während der Inhaftierung ein Testangebot gemacht und im Anschluss sichergestellt werden, dass ein positives Ergebnis nicht in der Gefangenenpersonalakte vermerkt wird. Ein Vermerk bezüglich einer Infektionsgefahr bei Blutkontakt kann zu Stigmatisierung der Betroffenen auf der einen und zu einem unberechtigten Sicherheitsgefühl der Mitarbeiter*innen auf der anderen Seite führen. Darüber hinaus sollten unabhängige Gesundheitsberatungen in Haft durch externe Anbieter*innen erfolgen, ggf. auch mit der Möglichkeit einer HCV-Testung sowie von Aktionswochen, in denen sich Gefangene freiwillig testen lassen können. Letzteres könnte zudem zu einer flächendeckenden Aufklärung und Beratung über die Behandlung und die damit einhergehenden Nebenwirkungen beitragen. Um die Behandlungsprävalenz in JVAen zu erhöhen, könnte es laut Expert*innen zielführend sein, wenn flächendeckend unabhängige Anstaltsärzt*innen eingesetzt und damit einhergehend die Kosten der Behandlungen von der GKV anstatt über die finanziellen Mittel der Justiz übernommen werden würden. Solange dies nicht umsetzbar ist, sollten sich Anstaltsärzt*innen proaktiver für eine Testung und anschließende Behandlung einsetzen und Angebote geschaffen werden, welche die Mehrfachbelastungen von Patient*innen in Haft berücksichtigen und adressieren. Somit könnte die Grundlage dafür geschaffen werden, dass Gefangene bereit sind, eine HCV-Behandlung innerhalb der Haft zu beginnen.

Das auch international Handlungsbedarf besteht, zeigt ein 2021 veröffentlichtes Review [20]. So haben von 124 Ländern mit HCV-Strategien nur 28 spezifische Pläne zur Minimierung der HCV-Prävalenz in Haft. Als potenzielle Lösungen genannt werden unter anderem schnellere Diagnosen durch *Point of Care Testing* (ermöglicht die Probenentnahme durch Fingerstich oder Speicheltestung und eine direkte Auswertung der Probe vor Ort), *Peer-Support*-Gruppen (um das Stigma um HCV zu verringern) und die Vorbereitung vor Haftentlassung im Kontext von HCV-Prävention. Relevant für die aktuelle Situation in Deutschland ist auch, dass die Autor*innen des Reviews die Bedeutung flächendeckend erhobener Daten zur HCV-Prävalenz in Haft besonders hervorheben: Ohne diese Daten fehle oft der politische Wille für eine gezielte Strategie [20, 21]. In Australien wurden jüngst Richtlinien des *National Prison Hepatitis Network* der Regierung vorgelegt, die flächendeckende Tests, schnelle Diagnostik und die Vergabe von Konsummaterialen sowie eine Intensivierung der Opioidsubstitutionstherapie fordern [22].

Aktuell startet in NRW und Hessen ein in einer länderübergreifenden Arbeitsgruppe entwickeltes Modellprojekt, das Wege aufzeigen soll, wie die HCV-Behandlungsquoten durch Standards im Bereich der Prävention, Diagnostik und Therapie erhöht werden können [20]. Um die Eliminierungsziele der UN zu erreichen, muss in einem integrierten Vorgehen auch die Situation in Haft mitbedacht werden und neben einer Ausweitung des Test- und Behandlungsangebotes der Fokus auf die Bereitstellung indirekter und direkter infektionsprophylaktischer Maßnahmen gelegt werden. Hierfür sollten Bund, Länder und Kommunen gemeinschaftlich und evidenzbasiert handeln – sowohl was die Bedingungen in Haft angeht als auch außerhalb.
